# Restoring functional TDP-43 oligomers in ALS and laminopathic cellular models through baicalein-induced reconfiguration of TDP-43 aggregates

**DOI:** 10.1038/s41598-024-55229-9

**Published:** 2024-02-26

**Authors:** Hsiang-Yu Chang, I-Fan Wang

**Affiliations:** 1 Garage Brain Science, B201, Central Taiwan Innovation Campus, Ministry of Economic Affairs, Nantou City, 540219 Taiwan; 2 Yee Fan Med Inc, Temple City, CA 91780 USA; 3 SABNP Lab, Univ Evry, INSERM U1204, Université Paris-Saclay, 91025 Evry, France

**Keywords:** Baicalein, TDP-43, Physiological oligomers, FTLD-U, ALS, Low complexity (LC) domain, Aggregates, Hutchinson-Gilford progeria syndrome (HGPS), Phenotypic screening, Ageing

## Abstract

A group of misfolded prone-to-aggregate domains in disease-causing proteins has recently been shown to adopt unique conformations that play a role in fundamental biological processes. These processes include the formation of membrane-less sub-organelles, alternative splicing, and gene activation and silencing. The cellular responses are regulated by the conformational switching of prone-to-aggregate domains, independently of changes in RNA or protein expression levels. Given this, targeting the misfolded states of disease-causing proteins to redirect them towards their physiological conformations is emerging as an effective therapeutic strategy for diseases caused by protein misfolding. In our study, we successfully identified baicalein as a potent structure-correcting agent. Our findings demonstrate that baicalein can reconfigure existing TDP-43 aggregates into an oligomeric state both in vitro and in disease cells. This transformation effectively restores the bioactivity of misfolded TDP-43 proteins in cellular models of ALS and premature aging in progeria. Impressively, in progeria cells where defective lamin A interferes with TDP-43-mediated exon skipping, the formation of pathological TDP-43 aggregates is promoted. Baicalein, however, restores the functionality of TDP-43 and mitigates nuclear shape defects in these laminopathic cells. This establishes a connection between lamin A and TDP-43 in the context of aging. Our findings suggest that targeting physiological TDP-43 oligomers could offer a promising therapeutic avenue for treating aging-associated disorders.

## Introduction

TDP-43 is a highly prevalent nuclear protein that plays a critical role in numerous biological processes, such as controlling polymerase II-dependent transcription, pre-mRNA splicing, microRNA biogenesis, and protein translation. Its functions also extend to vital cellular activities like neurite outgrowth, axonal transport, cell cycle regulation, and apoptosis^1–9^. The majority of TDP-43 proteins primarily reside in the nucleus and constantly shuttle between the nucleus and cytosol, ensuring the proper transport of RNAs (2).

A significant feature of TDP-43 is the presence of a prion-like low domain (PLD) at its C-terminus^10^. Under misfolded conditions, this domain becomes ubiquitinated and phosphorylated, leading to the accumulation of toxic aggregates in the brains of patients with various neurodegenerative diseases, including frontotemporal lobar degeneration with limbic-predominant age-related TDP-43 encephalopathy (LATE), ubiquitin-positive inclusions (FTLD-U) and amyotrophic lateral sclerosis (ALS), Alzheimer’s disease (AD), and Parkinson’s disease (PD)^11–18^. The discovery of around 30 ALS-causative mutations located in the C-terminal region of TDP-43 further supports its direct involvement in disease etiology^19^. Under physiological condition, the prion-like characteristics of TDP-43 enable it to undergo self- and hetero-polymerization, crucial for its various cellular functions^10^. These include the assembly of nuclear subdomains with diameters of 50–250 nm or irregular puncta, exon skipping in the cystic fibrosis transmembrane conductance regulator (CFTR), and the enhancement of protein stability and TDP-43 localization^10^. Despite an increasing number of studies suggesting that these prion-like polymeric forms play crucial roles in biological reactions, further in-depth investigation is necessary to fully understand the exact characteristics and biological importance of physiological TDP-43 oligomers.

In this study, we demonstrated that baicalein, a compound found in certain plants, most notably in the root of *Scutellaria baicalensis*, is capable of remodeling existing TDP-43 aggregates into polymeric states in vitro. This process also restores the functional activity of misfolded TDP-43 proteins in cell-based models of ALS and premature age. These compelling findings underscore the therapeutic promise of producing physiological TDP-43 oligomers from misfolded TDP-43 as a means of intervention. This approach holds significant potential for addressing neurodegenerative disease and aging.

### Baicalein, an off-amyloid pathway compound, remodels natively unfolded monomers and misfolded TDP-43 fibers into TDP-43 polymers in vitro and in vivo

**Figure 1:**
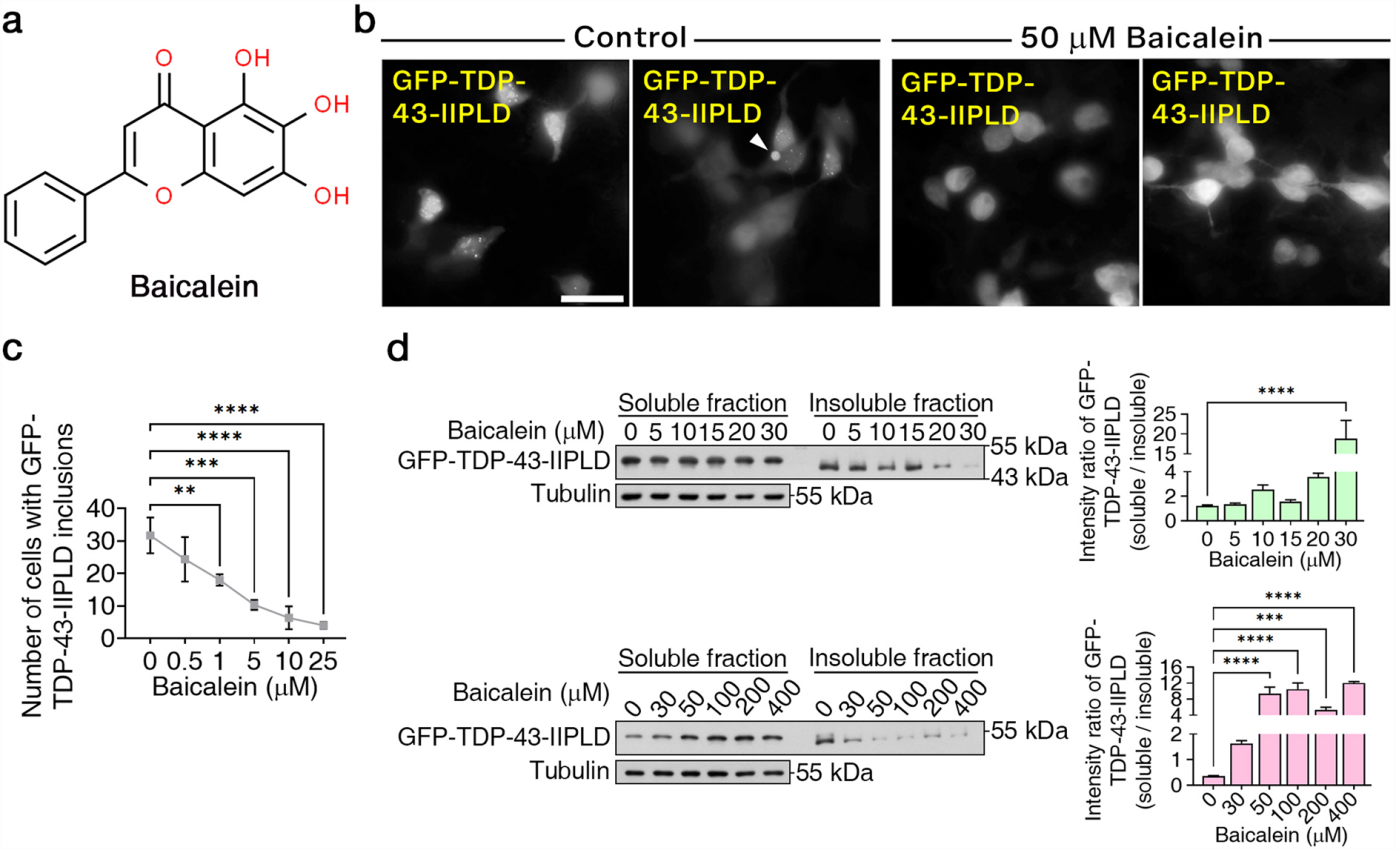
Baicalein remodeled misfolded TDP-43 into oligomers in vitro. (**a**) The chemical structure of baicalein. (**b**) 293 T cells with GFP-TDP-43-IIPLD were treated with 50 μM baicalein or no baicalein. Two images of individual treatments are shown. The arrowhead indicates TDP-43-IIPLD aggregates. Bars: 10 μm. (**c**) The statistical analysis of the effect of baicalein on GFP-TDP-43-IIPLD aggregates is shown. All data are presented as the means with SD (n = 3) and analyzed by one-way ANOVA. **P < 0.01. ***P < 0.001. ****P < 0.0001. (**d**) Western blotting analysis of the effectiveness of baicalein at enhancing the solubility of GFP-TDP-43-IIPLD post-treatment with baicalein for 9 h, respectively. All data are presented as the means with SD (n = 3) and analyzed by one-way ANOVA. **P < 0.01. ***P < 0.001. ****P < 0.0001.

In our endeavor to identify a compound capable of disassembling TDP-43 aggregates, we conducted a meticulous screening of a compound library. The investigation revealed baicalein’s efficacy in reducing pathological-like inclusions of GFP-tagged TDP-43 (GFP-TDP-43-IIPLD which includes the RRMII domain and Prion-Like Domain) within 293 T cells. GFP-TDP-43-IIPLD expressing 293 T cells were incubated with or without 50 μM baicalein for 12 h, followed by microscopic analysis and Western blotting validation. The chemical structure of baicalein is shown in Fig. 1a. As shown in Fig. 1b, baicalein inhibited the assembly of proteinaceous GFP-TDP-43-IIPLD nucleating particles (arrowhead indicates GFP-TDP-43-IIPLD aggregates). A statistical analysis showed a dosage-dependent reduction of GFP-TDP-43-IIPLD aggregates by baicalein (Fig. 1c). Consistently, Western blotting showed a reduction of insoluble GFP-TDP-43-IIPLD proteins in baicalein-treated cells (Fig. 1d). Cells exhibited a significant compensatory increase in the soluble fraction, suggesting that baicalein disassembled pathological TDP-43 aggregates instead of promoting degradation (Fig. 1d).

**Figure 2:**
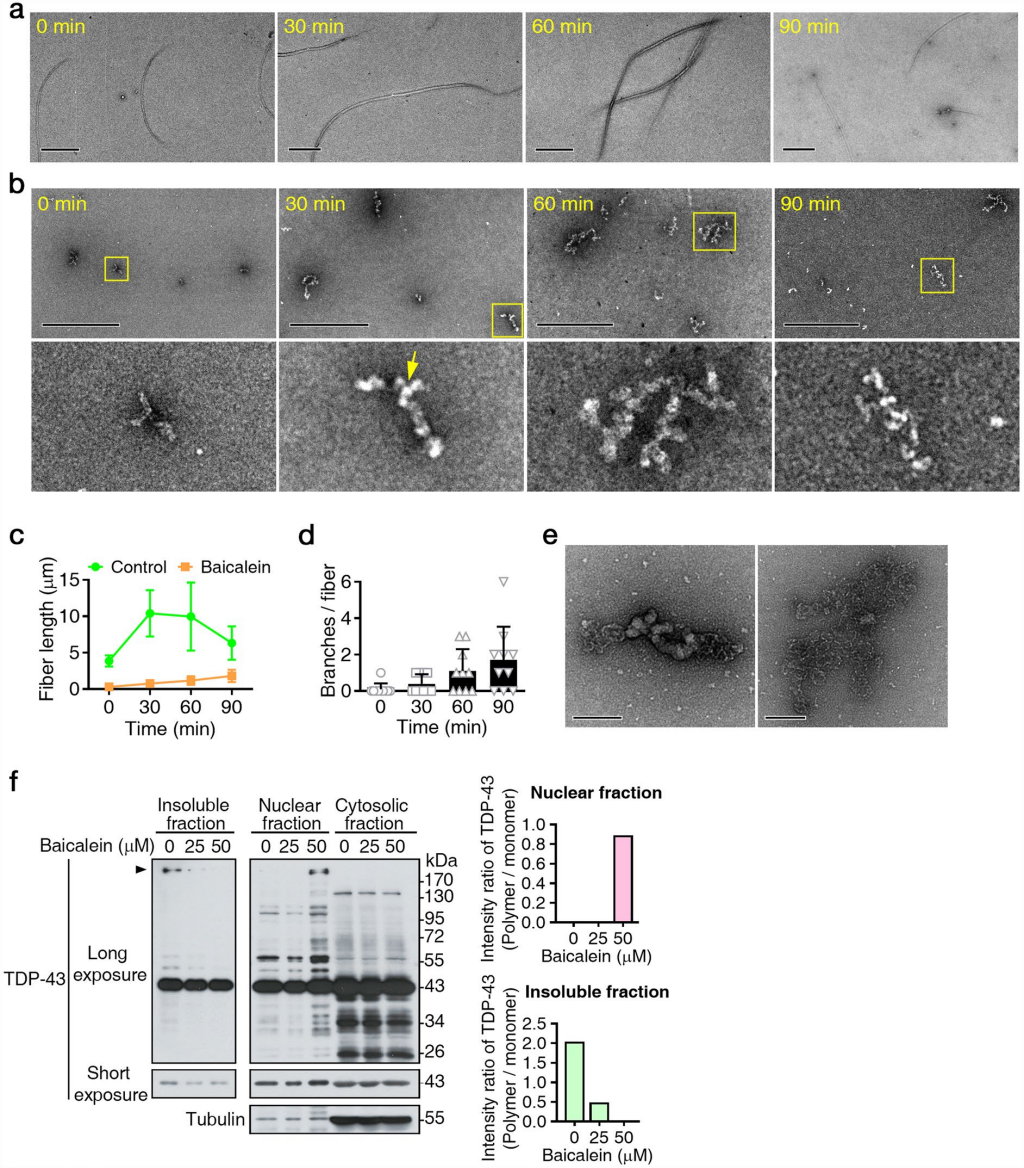
Baicalein remodeled misfolded TDP-43 fibers into oligomers in vitro and in vivo*.* (**a**,**b**) Purified full-length TDP-43 recombinant proteins (equivalent 3 μM monomer) were incubated in the absence (**a**) or presence of baicalein (3 μM; **b**) in assembly buffers at RT and agitated for 30 min, 60 min, and 90 min followed by validation with electron microscopy. Square frame in (**b**) indicate representative high-magnification images of TDP-43 oligomers in the lower panel. One of the branching points is indicated with an arrow. Bars in d: 1 μm; Bars in e: 0.5 μm. (**c**) Statistical analysis of TDP-43 fiber and oligomer length. All data are presented as the means with SD (n = 6). (**d**) Branching point analysis of baicalein-induced TDP-43 polymers. All data are presented as the means with SD (n = 10). (**e**) Two selected electron micrographs of negatively stained polymerizing TDP-43 structures. Scale bars: 100 nm. (**f**) Western blotting analysis of TDP-43 proteins with or without baicalein. 293 T cells were treated with 0, 25, or 50 μM baicalein following separation into nuclear, cytosolic, and insoluble fractions. Arrowhead indicates TDP-43 polymers.

Next, we incubated purified TDP-43 recombinant proteins with or without the compound (equimolar concentration) in assembling buffers at RT for 0, 30, 60, and 90 min with agitation, followed by an examination with negative staining electron microscopy. TDP-43 recombinant proteins formed fibers, and the length of TDP-43 fibers gradually increased from 3 to 10 μm (Fig. 2a). Interestingly, in the presence of baicalein, TDP-43 fibers, oligomers or natively unfolded monomers were efficiently remodeled into ordered TDP-43 polymers, in which TDP-43 proteins time-dependently accumulated as globular structures (approximately 30 nm long) along a string (Fig. 2b). Square frames indicate representative high-magnification images of TDP-43 oligomers. The baicalein-induced TDP-43 oligomers were 0.15–0.9 μm long and considerably shorter than the TDP-43 fibers. The length of the TDP-43 fibers and TDP-43 oligomers at different time points was analyzed (Fig. 2c). Notably, unlike tubular structures such as protofibers, these baicalein-induced TDP-43 oligomers had a highly branched structure, and the branches gradually increased to form ordered expressing 293T cells were incubated with or without 50 μM with increasing reaction times (Fig. 2d). One of these branching points is indicated by the arrow in the lower panel of Fig. 2b. Figure 2e shows two selected images of polymerizing TDP-43. These results indicated that baicalein directly binds to TDP-43 and transform the misfolded aggregated state into TDP-43 oligomers in vitro.

Accordingly, we investigated the pharmacological action of baicalein by analyzing the TDP-43 protein species present in baicalein-treated cells. We observed that TDP-43 oligomers significantly reduced the insoluble-fraction in baicalein-treated cells, yielding a compensatory increase in the nuclear fraction (Fig. 2f, arrowhead). Accordingly, we observed a functional correlation between nuclear TDP-43 oligomers and TDP-43 mediated exon skipping of CFTR through the manipulation of HSPB1 expression (Fig. S1). These results of HSPB1 provide a line of evidence for nuclear TDP-43 oligomers promotes exon skipping of CFTR^20^.

### Baicalein rescues TDP-43 dysfunction in disease cells of ALS

**Figure 3:**
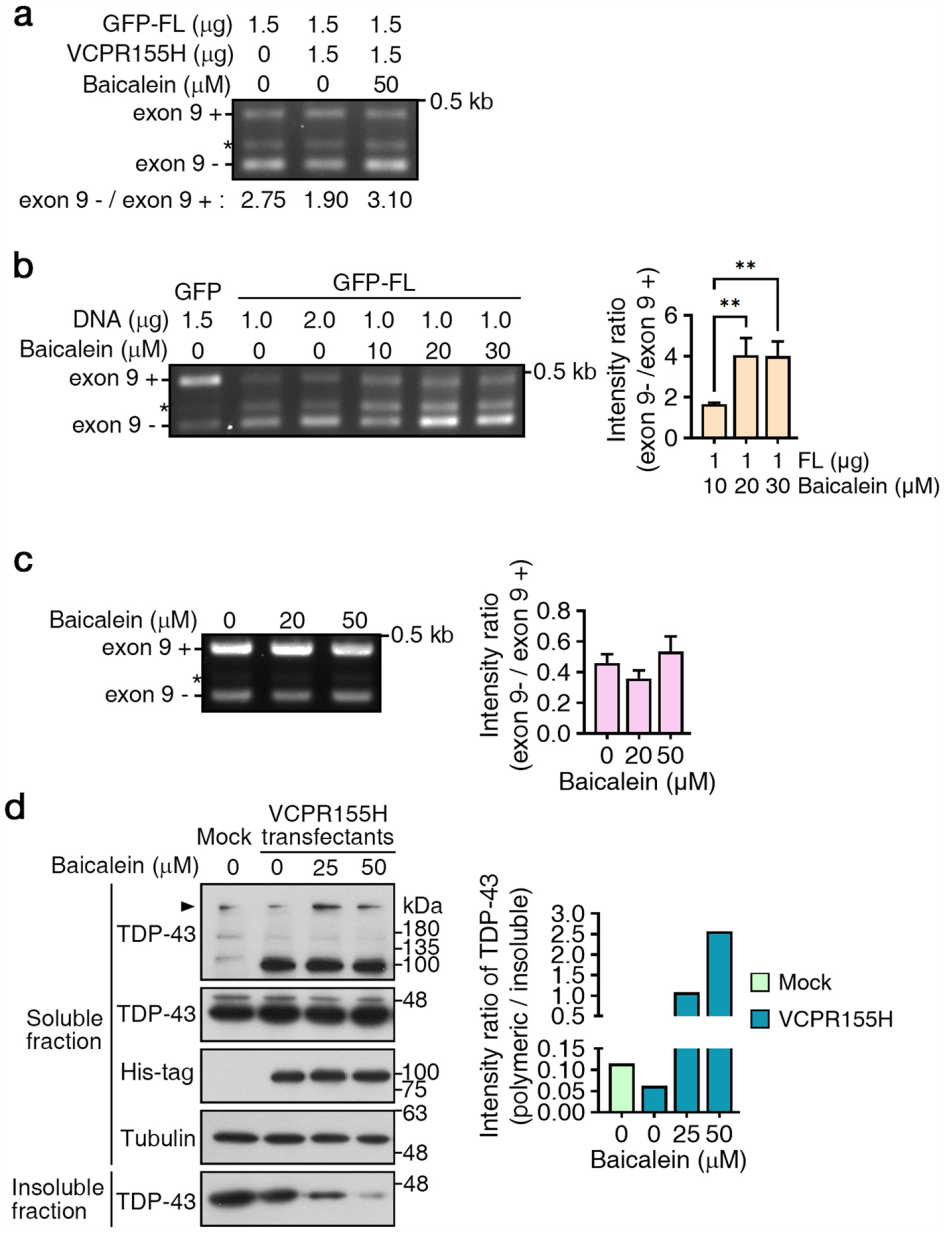
Baicalein rescued TDP-43 dysfunction in an inherited VCP97 mutation cell-based model of ALS. (**a**) Examination of TDP-43 alternative splicing ability in the presence of the VCP/p97 mutant R155H with or without baicalein by an in vivo splicing assay. Exon 9 inclusion (+) and exclusion (−) bands are indicated. *Aberrant splicing product. (**b**) In vivo assay of TDP-43-mediated CFTR exon 9 skipping in cells treated with or without baicalein. **P < 0.01 by ANOVA. (**c**) Examination of the effect of baicalein on CFTR exon 9 skipping in the absence of TDP-43 overexpression. (**d**) Western blot analysis of TDP-43 proteins in VCPR155H-expressing cells with or without baicalein. Arrowhead indicates TDP-43 polymers. (**e**) The dependent origination and fPLD-based therapeutic strategy of proteinopathy.

Next, we tested whether baicalein can functionally rescue the bioactivity of diseased TDP-43 proteins in cell-based disease model of ALS. Previously, TDP-43 proteinopathies in FTLD/ALS with the VCP/p97 mutation R155H have been characterized^21^. The VCP/p97 mutation R155H alters the function of VCP/p97, redistributing TDP-43 to the cytosol and leading to the formation of insoluble TDP-43 aggregates. Because functional TDP-43 proteins promote CFTR exon 9 skipping, we used an in vivo splicing assay to validate the folding state of cellular TDP-43 proteins in the presence of baicalein (Fig. 3a). In cells co-transfected with TDP-43 and the VCP/p97 R155H mutant, TDP-43 failed to promote CFTR exon 9 skipping. Significantly, this failure was rescued in the presence of baicalein (Fig. 3a). Cells treated with only baicalein showed an enhanced ability to promote TDP-43-mediated CFTR exon 9 skipping (Fig. 3b). If TDP-43 proteins were not overexpressed, baicalein had no effect on CFTR exon skipping (Fig. 3c), confirming that the effect of baicalein on CFTR exon skipping is TDP-43-dependent. As expected, a correlation between the rescue of TDP-43-mediated exon skipping of CFTR and the appearance of increased soluble nuclear TDP-43 oligomers was also observed in baicalein-treated VCP/p97 R155H cells (Fig. 3d, arrowhead), demonstrating that baicalein functionally rescued TDP-43 disease proteins in a VCP/p97 mutation-induced disease model by producing nuclear TDP-43 oligomers.

### An aging-associated mutation in lamin A leads to TDP-43 proteinopathy counteracted by baicalein

**Figure 4:**
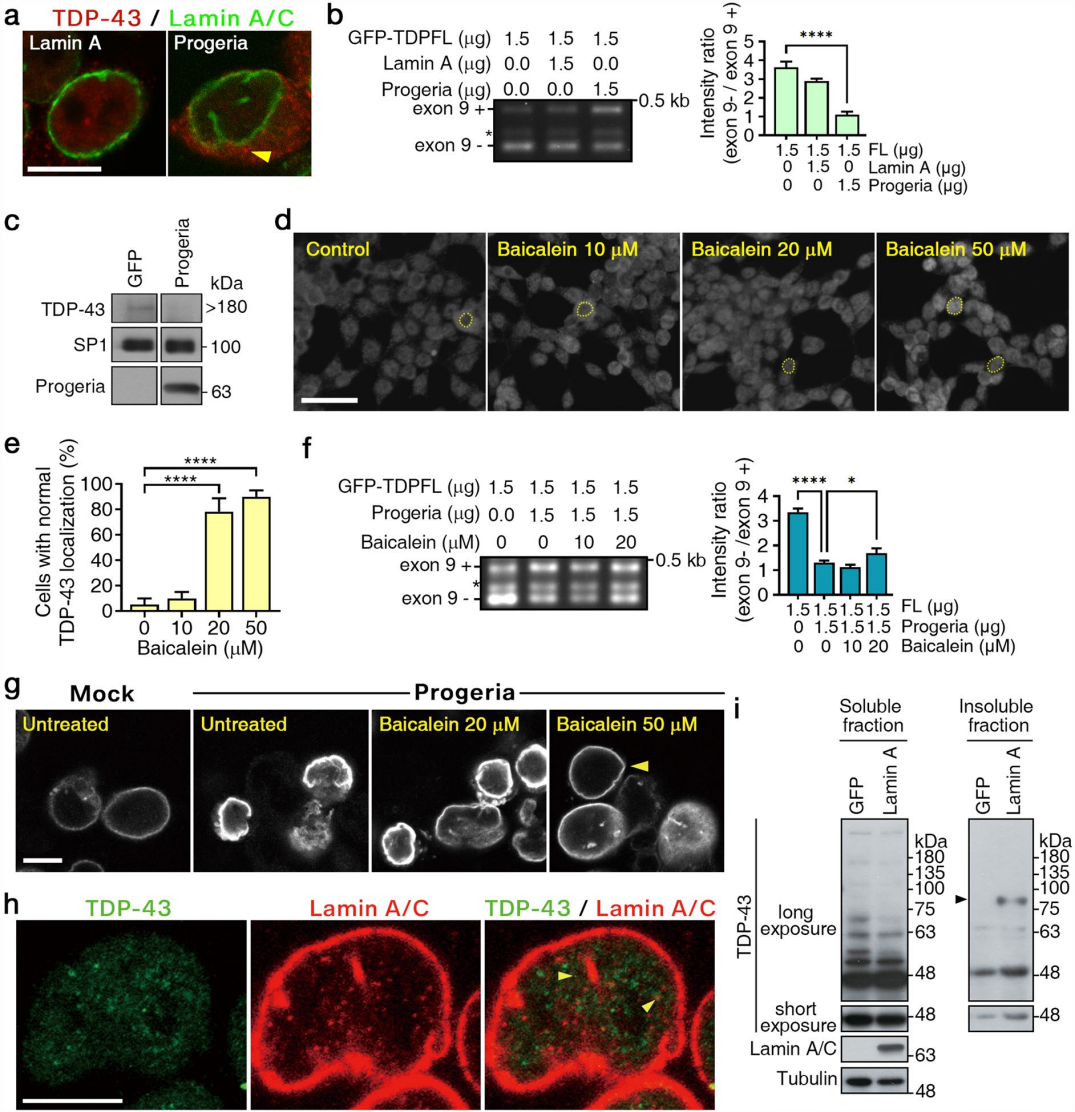
Baicalien rescued TDP-43 dysfunctions in Hutchinson-Gilford progeria syndrome. (**a**) Localization of GFP-TDP-43-FL proteins in lamin A- and progeria-expressing cells. Arrowhead indicates cytosolic aggregates of TDP-43. Scale bars: 10 µm. (**b**) Examination of TDP-43 alternative splicing ability in the presence of lamin A or progeria by an in vivo splicing assay. Exon 9 inclusion ( +) and exclusion (−) bands are indicated. *Aberrant splicing product. ****P < 0.0001 by ANOVA. (**c**) Western blotting for the validation of TDP-43 polymers in progeria-expressing cells. (**d**) Localization of TDP-43 in cells expressing progeria with or without baicalein. Bars: 10 μm. The cell nucleus is indicated by a dashed line. (**e**) The statistical analysis of baicalein rescue of TDP-43 nuclear localization is shown. All data are presented as the means with SD (n = 3). ****P < 0.0001 by by one-way ANOVA. (**f**) Examination of TDP-43 alternative splicing ability in the presence of baicalein in cells expressing progeria by an in vivo splicing assay. Exon 9 inclusion (+) and exclusion (−) bands are indicated. *Aberrant splicing product. (**g**) Immunostaining of nuclear shape in progeria-expressing cells with or without baicalein using anti-lamin A/C antibodies. Arrowhead in b indicates misshapen nuclei, and arrowhead in c shows rescued nuclei Bars: 10 μm. (**h**) Immunostaining of TDP-43 and lamin A/C. Green: TDP-43; Red: lamin A/C, Scale bars: 5 µm. (**i**) Western blotting for the validation of TDP-43 species in lamin A expressing cells.

Misfolded TDP-43 aggregates are commonly found in the brains of older adults over age 80 years, and have been linked to cognitive decline; however, it remains elusive whether a direct relationship exists between aging and TDP-43 proteinopathies^16,17^. As a lamin A mutantcan cause progeria, a premature aging disorder known as Hutchinson-Gilford Progeria syndrome (HGPS), overexpression of progeria proteins is considered a cell model of aging. This model allowed us to explore the mechanistic link between TDP-43 and aging or a lamin A pathology^22^. To explore this link, we overexpressed progeria proteins and examined TDP-43 localization, the efficiency of TDP-43PLD-mediated exon 9 skipping, and TDP-43 oligomers. In progeria-expressing cells, we observed that TDP-43 proteins exhibited a pattern of diffusion and cytoplasmic mislocalization and failed to promote CFTR exon 9 skipping (Fig. 4a,b, respectively). Significantly, in a few cells, we also observed cytosolic aggregates of TDP-43, which is a pathological hallmark of LATE and FTLD (Fig. 4a, arrowhead). Western blotting further revealed the assembly of TDP-43 oligomers in progeria-expressing cells is less efficient (Fig. 4c). We further investigated whether baicalein could rescue the progeria-induced dysfunction of TDP-43. As shown in Fig. 4d, baicalein significantly induced the retention of nuclear TDP-43 in cells expressing progeria proteins. A statistical analysis is presented in Fig. 4e. An in vivo splicing assay revealed that baicalein rescued TDP-43 dysfunction caused by progeria manner (Fig. 4f). The ratio of exon skipping restoration by baicalein ranged from 1.31 to 1.91 with a baicalein dosage of 10 μM to 50 μM (the ratio is indicated at the bottom of Fig. 4f). These results indicate that the progeria mutation disturbs TDP-43 PLD-mediated alternative splicing, possibly due to a failure in TDP-43 oligomeric assembly. However, these TDP-43 dysfunctions can be functionally corrected by baicalein. Additionally, we noticed that baicalein not only restored the activity of TDP-43 but also corrected nuclear shape defects in HGPS (Fig. 4g; arrowhead indicates rescued nuclei).

To explore the association of TDP-43 proteins with the nuclear matrix, which comprises proteins like lamin A/C, we conducted double-staining of TDP-43 (displayed in green) and lamin A/C (shown in red) (Fig. 4h). The staining indicated that TDP-43 proteins show partial colocalization with lamin A/C (see Figs. 4h and S2; the arrow highlights the area of colocalization). Nonetheless, even with the observed partial colocalization in puncta via immunofluorescence, we couldn’t identify a direct physical interaction between TDP-43 and lamin A/C using coimmunoprecipitation. However, we detected a notable change in TDP-43 species in cells expressing lamin A (Fig. 4i). Specifically, the 54 kD and 70 kD TDP-43 species were absent, while there was a noticeable assembly of insoluble 90 kD TDP-43 proteins (indicated by an arrowhead) in cells with overexpressed lamin A (Fig. 4i). This data infers that lamin A levels can modulate the post-modification, tertiary structure formation, and even the solubility of TDP-43.

## Discussion

In our earlier research, we pinpointed a GQN-rich domain within the non-amyloid protein TDP-43^10^. Remarkably, this domain could be swapped functionally with the yeast prion domain Sup35N14. Such a discovery suggests that both amyloid and non-amyloid disease proteins exhibit a similar cellular folding pattern when in healthy cells, predisposing them to condensation^10^. This shared folding configuration offers a promising avenue for treating TDP-43 proteinopathies with off-amyloid stabilizers. To evaluate this possibility, we explored a range of off-amyloid stabilizers from a compound library. Baicalein emerged as a leading candidate^23,24^. Not only did it disassemble TDP-43 fibers both in vivo and in vitro, but it also rehabilitated misfolded TDP-43 to its functional form in cellular disease models. Furthermore, a detailed pharmacological assessment of baicalein divulged new understandings of its mechanism of action. This revealed the existence of an oligomeric form of TDP-43, which plays a role in TDP-43-driven alternative splicing and is linked to age-related TDP-43 pathology. Recent studies have highlighted the significance of polymeric prion-like domains in regulating essential cellular functions such as transcription, RNA processing, and DNA repair^25–29^. For instance, TAF15 fibrous polymers interact with the C-terminal domain (CTD) of RNA polymerase II, influencing transcriptional activation^26^. Similarly, the Fus protein forms aggregates that play a pivotal role in maintaining genomic stability in the face of DNA damage^29^.

We postulate that altering the folding states of disease-causing proteins towards their active states might simultaneously address various misfolded protein pathologies. This includes symptoms stemming from both gain- and loss-of-function mutations of the disease proteins, prion-like spreading, and off-target effects. In the context of TDP-43 pathology, restoring the physiological functions of TDP-43 is paramount for therapeutic efficacy. This is because the loss of TDP-43 function can result in cell cycle abnormalities and induce neurodegeneration in organisms like flies, fish, and rodents^30,31^. Moreover, defective TDP-43 has been demonstrated to disrupt pre-mRNA alternative splicing and cause misregulation of transposable elements in patients with TDP-43 pathology^32,33^. The compound baicalein, highlighted in this study, has proven effective in counteracting TDP-43 proteinopathies.

Baicalein has been shown to effectively mitigate proteinopathies in mouse models of neurodegenerative diseases^34^. While it has been suggested to act through various mechanisms such as reducing oxidative stress and anti-inflammation effects, our study identifies an additional and perhaps primary mode of action, refolding misfolded proteins. Unfortunately, current mouse models for TDP-43 proteinopathies are inadequate for assessing the therapeutic efficacy of baicalein-mediated refold of TDP-43. These models rely solely on the overexpression of mutated TDP-43, which poses two significant limitations. First, eradicating the surplus of TDP-43 proteins throughout the mouse’s life would require prohibitively high doses of baicalein. Second, the constant overexpression of the disease-specific protein can impede the drug's ability to neutralize the harmful effects of the protein, potentially yielding false negatives in terms of drug efficacy. Notably, this chronic overproduction of TDP-43 proteins is not a feature observed in human patients with TDP-43 proteinopathies. Future research should employ animal models that more accurately represent the human condition. Furthermore, to optimize baicalein's effectiveness in treating misfolded proteins, we proposed administering it during the preclinical stage, prior to the onset of symptoms. This allows for the timely refolding of these proteins. Thus, developing a preclinical diagnostic method is vital for accurately gauging baicalein’s treatment potential and may even pave the way for preventing neurodegenerative diseases.

## Materials and methods

### Chemical structure

The chemical structure of baicalein was illustrated by ChemSpider (CSID:4444924, http://www.chemspider.com/Chemical-Structure.4444924.html (accessed 11:16, Nov 26, 2023)).

### In vitro analysis of TDP-43 fiber formation

Three-micromolar full-length TDP-43 recombinant protein (GenWay) was incubated with 3 μM baicalein in an assembly buffer. The reactions were performed for 30 min, 60 min, and 90 min with agitation at RT. The resulting samples were stained by 4% uranyl acetate for 1 min. EM analysis was performed with a FEI Tecnai G2 Spirit TWIN transmission electron microscope.

### Cell culture and drug treatment

293 T cells were purchased from ATCC and maintained in Dulbecco’s modified Eagle’s medium (DMEM)/F12, which was supplemented with 10% fetal bovine serum, 1% penicillin/streptomycin. To determine the effects of off-pathway stabilizers on the reduction of pathological-like aggregates, the cells were treated with GFP-TDP-43-IIP plasmids for 12, 24, or 48 h with the indicated concentrations (25 or 50 μM) of baicalein.

### Plasmid constructs, siRNA and transfections

VCP/p97 wt constructs were generously provided by Dr. Yihong Ye (NIH/NIDDH). VCPR155H was further generated by a site-directed mutagenesis protocol (Qiagene). Plasmids encoding the wt or progerin form of lamin A were provided by Dr. Ya-Hui Chi (NHRI, Taiwan). The human HSPB1 gene was cloned into the pEGFP-N3 (Clontech, Mountain View, CA, USA) in a similar manner as in Wang et al.^10^. SiRNAs for HSPB1 studies were purchased from Santa Cruz Biotechnology (SC-29350). For the overexpression or knockdown experiments, individual plasmids or siRNA (20 or 40 pmol) were transiently transfected into 293 T cells according to the manufacturer’s guidelines for Lipofectamine 2000 reagent (Invitrogen). The GFP-TDP-43-IIPLD plasmid expressing a pathological-like fragment (a.a. 179–414, with RRMII and prion-like domains) was generated by amplifying the IIPLD fragmentfrom mouse cDNA using IIPLD primers, followed by its insertion into pEGFP-N3 (Clontech) using established protocols^10^.

### Reagents and antibodies

Baicalein was obtained from Sigma. Primary antibodies against HSPB1 were purchased from Cell Signaling Technology (Beverly, MA). The primary antibody against lamin A/C was purchased from Millipore Inc. The primary antibody against TDP-43 was purchased from Proteintech. Full-length images are included in Figs. S3 to S4. For blots cut prior to hybridization with antibodies, such as tubulin, we made the edges of these blots visible.

### Electron microscopy analysis

A 3-ml aliquot of the samples obtained following immunoprecipitation was adsorbed onto a glow-discharged 200-mesh copper grid covered with carbon-coated collodion film, washed with three drops of distilled water, and stained with two drops of 0.75% uranyl formate, and the samples were examined with a FEI Tecnai G2 Spirit TWIN transmission electron microscope.

### Immunofluorescence and image acquisition

For these assays, 293 T cells were transfected with or without plasmids and then fixed with 3.7% paraformaldehyde in PBS at RT for 15 min at 24 h (for GFP-TDP-43-IIPLD experiments) or 48 h post-transfection. The fixed cells were incubated with a TDP-43 or lamin A/C or His antibody at 4 °C overnight, followed by incubation with a secondary antibody conjugated with a fluorescent dye (Molecular Probes). The staining cells were gently fixed with 3.7% paraformaldehyde for 2 min before mounting. Slides were mounted using Vectashield DAPI H-1200 (Vector Laboratories). Cellular fluorescence images were obtained from a single optical section using an LSM710 META laser confocal microscope (Zeiss) or ELYRA super-resolution microscope (Zeiss)^33^.

### Quantification and statistical analysis

cDNAs were quantified using Image J software. The graphs are plotted according to relative intensity. The statistical significance was calculated via t-test. The difference between groups was considered to be significant when P < 0.05.

### Alternative splicing assay

TDP-43-mediated CFTR exon 9 skipping assays were performed as previously described^10^. Briefly, cells were cotransfected with TDP-43 plasmid, hCF-(TG)_13_(T)_5_ minigenes and the indicated plasmids, including VCP/p97 wt, HSPB1, lamin A or progeria, or HSPB1 siRNA. Total RNA of transfectanted cells was isolated by TRIzol reagent (Invitrogen), and RT-PCR was carried out by Superscript III (Invitrogen) using specific primers Bra2 and a2-3 to amplify exons 8–10 of CFTR according to the manufacturer’s protocol^10^. The relative amounts of cDNA were validated on 1.3% agarose gels.

### Preparation of nuclear, cytosolic, and insoluble fractions

The cells were lysed on ice for 5 min with buffer A (10 mM Tris, 10 mM NaCl, 3 mM MgCl_2_, 0.5% (v/v) NP-40, and 0.5 mM DTT, pH 7.4) and fractionated via centrifugation at 16,000×*g* for 5 min at 4 °C. The supernatant is the cytosolic fraction. The pellet was then resuspended in buffer B (20 mM HEPES, 1.5 mM MgCl_2_, 0.4 mM EDTA, 20% glycerol [v/v], and 0.5 mM DTT, pH 7.9). Next, a 1/10 volume of 3 M KCl was added, and the resulting mixture was on ice for 30 min and then fractionated via centrifugation at 16,000×*g* for 30 min at 4 °C. The supernatant is the nuclear fraction. The resulted pellet containing nuclear and cytosolic insoluble proteins is further dissolved in 8 M urea/50 mM Tris, pH 8.0, becoming the insoluble fraction.

### Supplementary Information

Supplementary Figure1.

Supplementary Figure 2.

Supplementary Figure 3.

Supplementary Figure 4.

## Data Availability

Available upon reasonable request to ifanwang.gbs@gmail.com.
