# Supplementary figures and images for: Restoring functional TDP-43 oligomers in ALS and laminopathic cellular models through baicalein-induced reconfiguration of TDP-43 aggregates
Source: Sci Rep. 2024 Feb 26;14:4620. doi: 10.1038/s41598-024-55229-9 (PMC10897466; doi:10.1038/s41598-024-55229-9)

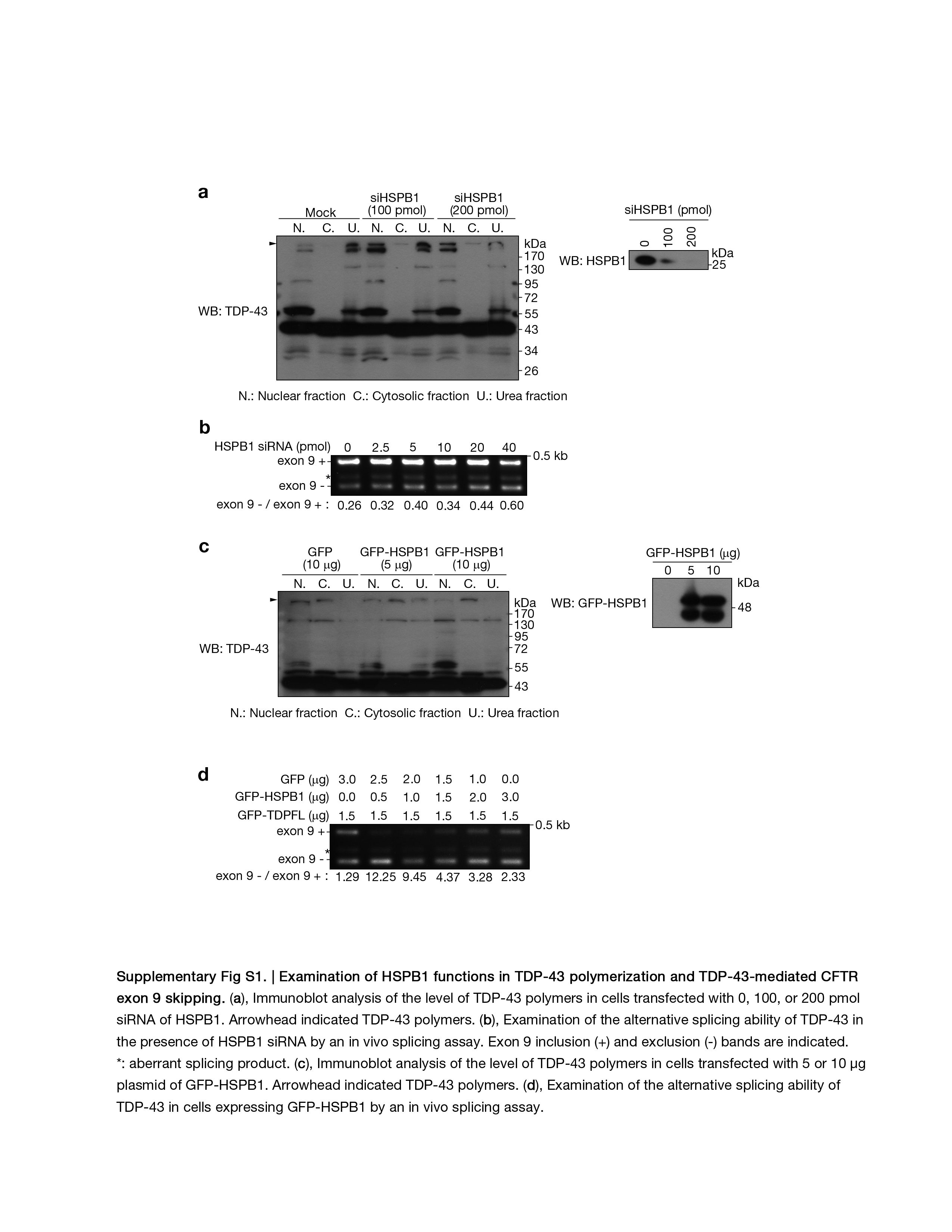

Supplement: Supplementary file 1 — Supplementary Figure1. [file 41598_2024_55229_MOESM1_ESM.jpg]

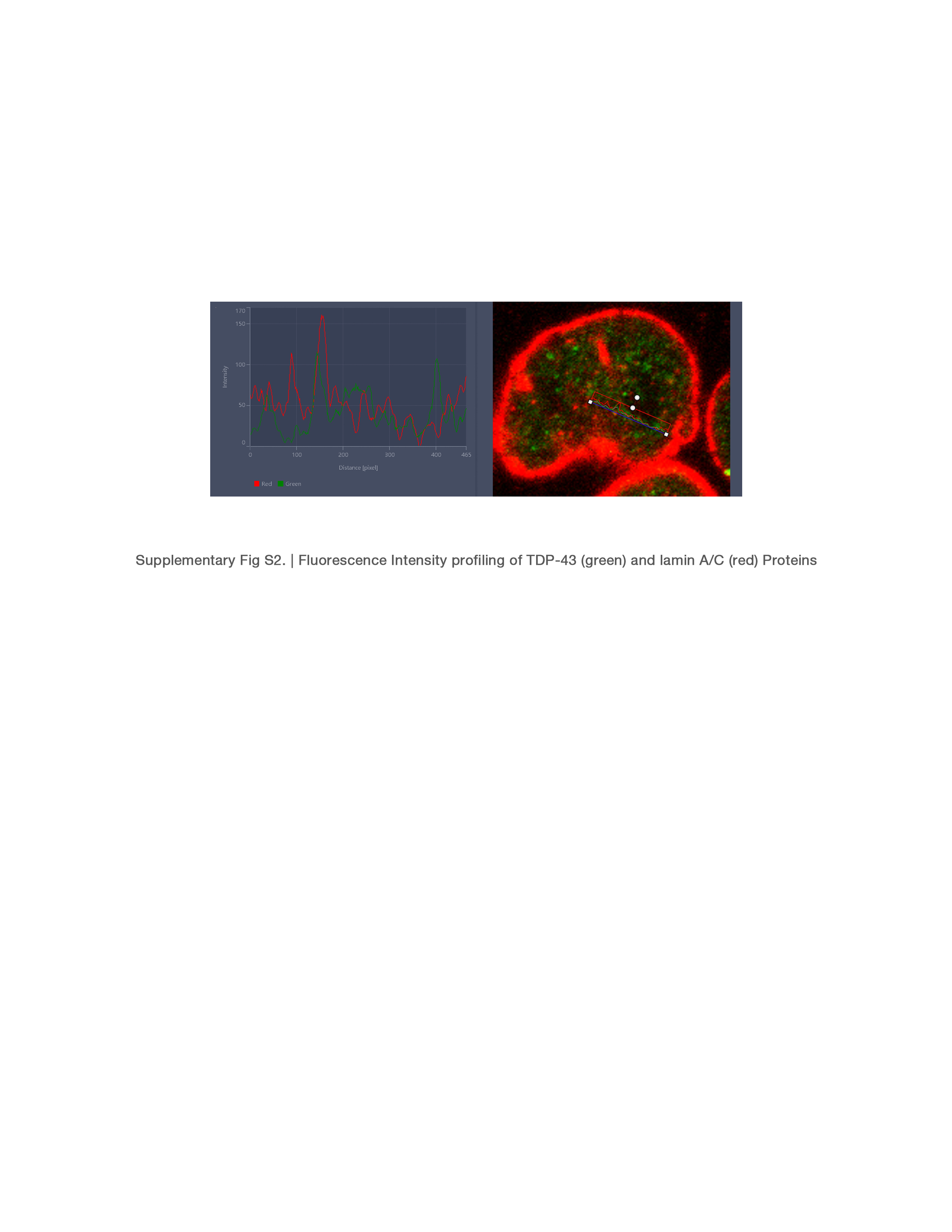

Supplement: Supplementary file 2 — Supplementary Figure 2. [file 41598_2024_55229_MOESM2_ESM.jpg]

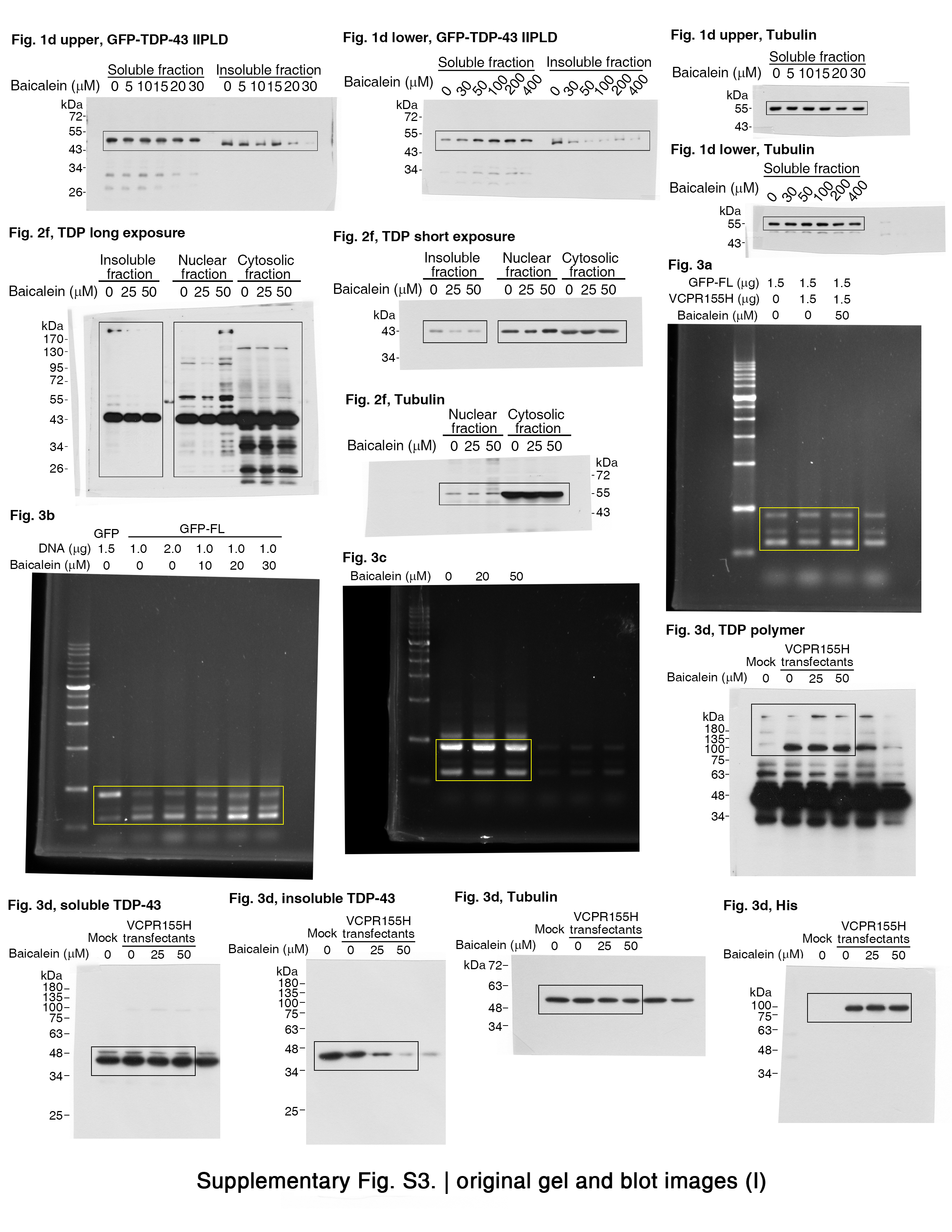

Supplement: Supplementary file 3 — Supplementary Figure 3. [file 41598_2024_55229_MOESM3_ESM.jpg]

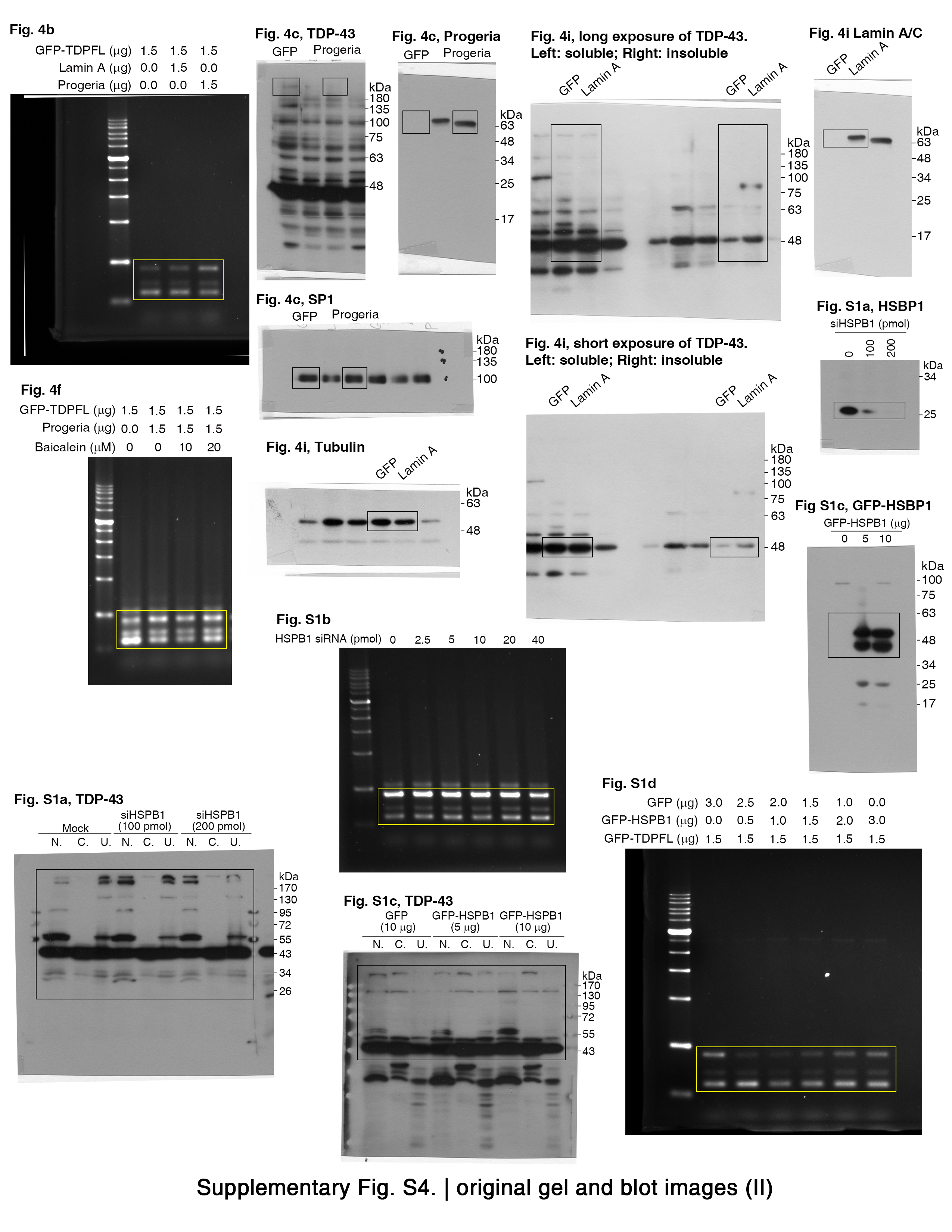

Supplement: Supplementary file 4 — Supplementary Figure 4. [file 41598_2024_55229_MOESM4_ESM.jpg]
